# A quantitative method for benchmarking fair income distribution

**DOI:** 10.1016/j.heliyon.2022.e10511

**Published:** 2022-09-03

**Authors:** Thitithep Sitthiyot, Kanyarat Holasut

**Affiliations:** a Department of Banking and Finance, Faculty of Commerce and Accountancy, Chulalongkorn University, Mahitaladhibesra Bld., 10th Fl., Phayathai Rd., Pathumwan, Bangkok, 10330, Thailand; b Department of Chemical Engineering, Faculty of Engineering, Khon Kaen University, Mittapap Rd., Muang District, Khon Kaen, 40002, Thailand

**Keywords:** Fairness, Income distribution, Inequality, Gini index, Sustainable Development Goals

## Abstract

Concern about income inequality has become prominent in public discourse around the world. However, studies in behavioral economics and psychology have consistently shown that people prefer not equal but fair income distributions. Thus, finding a benchmark that could be used to measure fair income distribution across countries is a theoretical and practical challenge. Here a method for benchmarking fair income distribution is introduced. The benchmark is constructed based on the concepts of procedural justice, distributive justice, and authority’s power in professional sports since it is not only widely agreed as an international norm that the allocations of athlete’s salary are outcomes of fair rules, individual and/or team performance, and luck but also in line with no-envy principle of fair allocation. Using the World Bank data, this study demonstrates how the benchmark could be used to quantitatively gauge whether, for a particular value of the Gini index, the income shares by quintile of a country are the fair shares or not, and if not, what fair income shares by quintile of that country should be. Knowing this could be useful for those involved in setting targets for the Gini index and the fair income shares that are appropriate for the context of each individual country before formulating policies towards achieving the Sustainable Development Goal 10 and other related SDGs.

## Highlights

• Inequality in income distribution is viewed as a serious concern worldwide
• However, studies show that people generally prefer fair allocations, not equal ones
• This study introduces a benchmark for measuring fair income distribution
• The benchmark is derived using the athletes’ salaries from 11 professional sports
• The income shares and the Gini index are used to show how the benchmark works

## Graphical abstract

**Figure:**
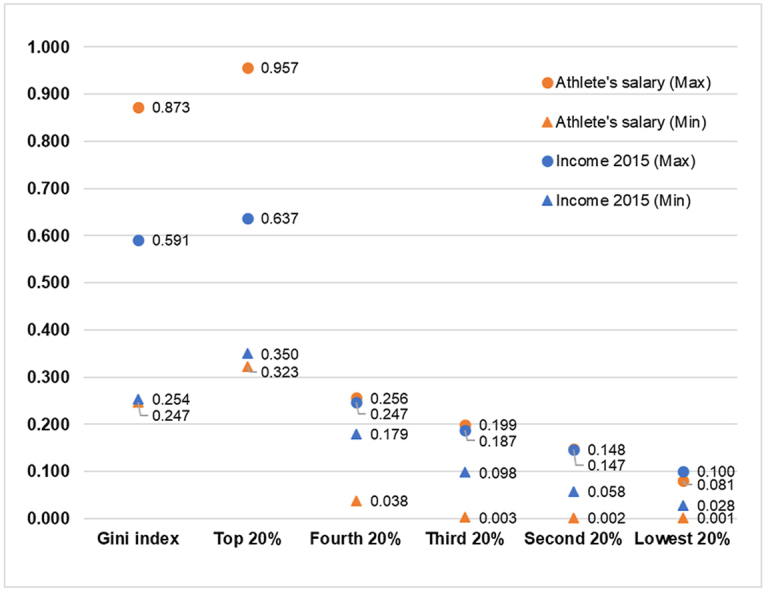

## 1 Introduction

Concern about inequality in income distribution has become prominent in public discourse around the world (Mayhew and Wills, 2019). According to the United Nations (2020a), in all countries with data, during the period 2012 to 2017, income is increasingly concentrated at the top where the richest 10% of the population receive at least 20% of the overall income while the bottom 40% receive less than 25% of total income. As income inequality increases, self-reported happiness diminishes, especially among the bottom 40% of the income earners (Oishi et al., 2011). In addition, income inequality predicts a greater degree of violence, obesity, teenage pregnancy, and interpersonal distrust (Wilkinson and Pickett, 2009). In the United States of America, areas with high income inequality tend to have higher divorce and bankruptcy rates than those with more equal income distributions (Frank et al., 2014) and they also suffer from higher homicide rates (Daly et al., 2001). According to the United Nations (2020b), high and rising inequality hinders progress towards the Sustainable Development Goals (SDGs). Reducing inequality within and among countries is regarded as one of the central themes of the 2030 Agenda for Sustainable Development. Target 10.1 of the SDG 10 calls for actions to reduce income-based inequality within countries by progressively achieving and sustaining income growth of the bottom 40% of the population at a rate higher than the national average by 2030 (the United Nations, 2020b).

While there is immense concern about income inequality, both among scholarly community and in general public, and many insist that equality is an important social goal (Starmans et al., 2017), research in behavioral economics, studying distributive behavior in situations where people have earned money being distributed, have found that the majority of people accept income inequalities as fair if the inequalities correspond to differences in contributions (Cappelen et al., 2014; Almås et al., 2020). Research in political psychology have also found that when people are asked about the ideal distribution of wealth in their country, they actually prefer unequal societies (Norton and Ariely, 2011; Kiatpongsan and Norton, 2014; Norton et al., 2014). Moreover, laboratory studies, cross-cultural research, and experiments with babies and young children have consistently shown that humans naturally favor fair distributions, not equal ones, and that when facing a choice between fairness and equality, people prefer *fair inequality to unfair equality* [emphasis added] (Starmans et al., 2017).

There are two concepts that are commonly accepted which could be used to justify whether or not inequality is seen as fair. The first concept states that people have to agree that inequality results from fair processes with regard to resource allocations (Trump, 2020). This is known as procedural justice where fairness is associated with processes by which the authorities enact rules, resolve disputes, and allocate resources (van Dijke et al., 2019). As long as people generally believe that the outcomes flow from fair allocation procedures, discontent would be muted (Tyler, 2011). Focusing on procedural justice is insufficient to justify whether inequality is viewed as fair, however. It is also relevant to consider the second concept which concerns how benefits and costs are distributed across individuals and/or groups. This is known as distributive justice where fairness is associated with the extent to which the outcomes of processes that allocate benefits and costs are perceived as matching distributive norms such as the equity rule which requires that individuals and/or groups should receive benefits and costs proportional to their contributions (van Dijke et al., 2019). According to van Dijke et al. (2019), studies have shown that people who perceive the distribution of outcomes across individuals, groups, and society as a whole as fair would react in more positive ways. Although inequalities produced through individual effort and merit are seen as fairer than those produced through luck (Almås et al., 2020), both adults and children agree that inequalities produced by using random allocation devices are acceptable (Karen and Teigen, 2010; Kimbrough et al., 2014; Shaw and Olson, 2014; Choshen-Hillel et al., 2015; Molina et al., 2019). In real life, people often disagree on what is fair because they disagree on whether individual achievements, luck, and efficiency considerations of what maximizes total benefits can justify inequalities (Almås et al., 2010). Thus, what considered as fair do vary across cultures and societies (Rochat et al., 2009; Schäfer et al., 2015; Almås et al., 2020; Apel, 2020). Focusing on the issue of fair income distribution, finding a quantitative benchmark that could be used as a reference for comparing income distribution across countries is therefore a theoretical and practical challenge. According to the Office of the United Nations High Commissioner for Human Rights (OHCHR) (2012), a meaningful benchmark should have a general consensus or an international norm on the choice of a candidate to be used for assessment.

Given the growing concern about income inequality among academic scholars and in general public on the one hand and the empirical evidence showing people’s preference for fair income distribution, not equal one on the other, it is important to find out whether the existing income distribution of a country is fair or not. If not, what should the fair distribution of income among population in that country be? While the political economy of what levels of income inequalities that countries would accept as fair remains an uncharted territory (Rogoff, 2012), to our knowledge, there have been very few studies that attempt to quantitatively measure how much inequality in income distribution is fair. These studies are Venkatasubramanian (2009, 2010, 2019), Venkatasubramanian et al. (2015), and Park and Kim (2021). Venkatasubramanian (2009, 2010, 2019) and Venkatasubramanian et al. (2015) develop a benchmark that is based primarily on the concept of maximum entropy in statistical mechanics and information theory, and propose that, in an ideal free market, fair income allocation should follow a log-normal distribution whereas Park and Kim (2021) propose the concept of a feasible income equality that maximizes the total social welfare and demonstrate that an optimal income distribution that represents the feasible equality could be modeled by using the sigmoid welfare function and the Boltzmann income distribution.

In this study, we introduce an alternative method for benchmarking fair income distribution. Our method is based on the concepts of procedural justice and distributive justice with regard to the allocations of athlete’s salary in the realm of professional sports. The justifications for choosing the allocations of athlete’s salary in professional sports are mainly because, according to Rogoff (2012), Brannon (2014), Kay (2015, p. 254), and Pritchett and Tiryakian (2020a; 2020b), it is widely agreed as an international norm that the allocations of athlete’s salary in professional sports are resulted from fair and transparent rules, individual and/or team effort, as well as luck, all of which fit well with the notions of procedural justice and distributive justice as discussed above. Professional sports are generally considered as a universal language since the rules of the games, the performances of the athletes, and the outcomes of the competitions are, by and large, understandable to and could be viewed and judged by ordinary people as fair, regardless of their origin, background, religious belief, or economic status. Brannon (2014) and Pritchett and Tiryakian (2020a; 2020b) note that few fans seem to begrudge the professional athletes for their money. This stylized fact has long been known in the fairness literature as no-envy principle of fair allocation which was introduced by the Nobel prize laureate in economics Jan Tinbergen in his article published in 1930. In line with no-envy principle of fair allocation, Harvard economist Kenneth Rogoff expresses his amazement over the public’s blasé acceptance of the salaries of sports stars compared to its low regard for superstars in business and finance (Rogoff, 2012). This is because it is easier to grasp the sacrifices an athlete in professional sport made to achieve stardom than a successful chief executive officer where vilification runs rampant (Brannon, 2014). The United Nations also recognizes the contribution of sports to the SDGs (the United Nations Office on Sport Development and Peace (UNOSDP), 2018). As stated by the UNOSDP (2018), sport value such as fairness can serve as an example for an economic system that builds on fair competition. In addition to the ideas of procedural justice and distributive justice, we follow van Dijke et al. (2019) by including the idea of power of authority as a basis to support our selection of professional sports for developing our method. van Dijke et al. (2019) define power of authority as a capacity to detect and punish misconducts and rule violations. According to van Dijke et al. (2019), high authority’s power is an important element of the process underlying the effects of procedural justice and distributive justice on voluntary rule compliance. This is similar to professional sports. When the power of sport authority is high, the athlete tends to voluntarily comply and adheres to the rules of the game.

Viewing through the lens of procedural justice, distributive justice, and authority’s power in professional sports, the distribution of athlete’s salary could thus be justified as a plausible candidate for developing a neutral benchmark for measuring fair income distribution since the salary is allocated based on the performance of each individual athlete (distributive justice) who competes according to fair and transparent rules (procedural justice) that are written and administered by the sports authority (power of authority), all of which, by and large, are comprehensible to and could be observed and judged by ordinary people around the world as fair as pointed out by Rogoff (2012), Brannon (2014), Kay (2015), the UNOSDP (2018), and Pritchett and Tiryakian (2020a; 2020b), and hence satisfies both the no-envy principle of fair allocation introduced by Tinbergen (1930) and the general consensus or the international norm criterion of a meaningful benchmark according to the OHCHR (2012). While this study proposes the use of the distribution of professional athlete’s salary for developing a benchmark for measuring fair income distribution, we are fully aware that other types of data could also be used if they are considered as fairer and more universally accepted than the data on the distribution of athlete’s salary in professional sports, provided such data can be numerically ranked.

To develop our benchmark, the data on the annual salaries of athletes from 11 well-known professional sports in 2019 or the latest from Sitthiyot (2021) comprising 6,709 observations are employed in order to construct the fairness lines. These fairness lines are derived from the statistical relationship between the salary shares of the athlete in each quintile and the Gini index for the athlete’s salary of 11 professional sports. They are the Women's National Basketball Association (WNBA), the English Premier League (EPL), the National Football League (NFL), the National Hockey League (NHL), the Major League Baseball (MLB), the National Basketball Association (NBA), the Professional Golfers' Association of America (PGA), the Ladies Professional Golf Association (LPGA), the Major League Soccer (MLS), the Association of Tennis Professionals (ATP), and the Women's Tennis Association (WTA).

It is very important to note that rules, regulations, and player’s codes of conduct in these professional sports are written and administered by relevant international sports authorities whose members are from different countries. In addition, the athletes competing in these professional sports come from diverse ethnic, social, national, and regional backgrounds and receive compensations based on their performances, skills, and luck. Furthermore, these professional sports tournaments, by and large, are taken place in different countries around the world. For these reasons, employing the data on the athletes’ salaries from 11 professional sports should avoid or mitigate the problem of selection bias since they do not come from one specific group of population and/or one particular country.

By using the annual data on the Gini index and the income shares by quintile in 2015 from the World Bank (2020a) containing 75 countries, this study shows how the fairness lines could be used as a benchmark to quantitatively gauge whether, for an existing value of a country’s Gini index, the income shares in each quintile of that country are the fair shares or not, and if not, what level of fair income shares in each quintile of that country should be. We also demonstrate that, by using our fairness benchmark, policymakers could choose a combination of the Gini index and the fair income shares by quintile that are appropriate for the context of that country. This could benefit whoever involved in formulating strategies and policies aimed to achieve not equal, but fair income distribution societies, the SDG 10, and other related SDGs. In addition, given that the fair income distribution depends on the country context and can vary considerably, our method could be used to compare fair income distribution among different groups within a country as well as across countries since they are measured using the same benchmark.

## 2 Materials and methods

For each of 11 professional sports, namely, WNBA, EPL, NFL, NHL, MLB, NBA, PGA, LPGA, MLS, ATP, and WTA, the derivation of the benchmark for fair income distribution begins by normalizing the annual salaries of the athletes and ranking them in an ascending order. We then plot the Cartesian coordinates where the abscissa is the cumulative normalized rank of athlete’s salary (x) and the ordinate is the cumulative normalized athlete’s salary (y). This would give us the actual Lorenz plot for each type of sports. Next, we fit the estimated Lorenz curve to the actual Lorenz plot for each type of 11 professional sports by employing a parametric functional form which has a closed-form expression for the Gini index developed by Sitthiyot and Holasut (2021). This specified functional form is constructed based on the weighted average of 2 well-known functional forms for estimating the Lorenz curve which are the exponential function $\left(\text{y}\left(\text{x}\right)={\text{x}}^{\text{P}}\right)$ and the functional form implied by Pareto distribution $\left(\text{y}\left(\text{x}\right)=1-{\left(1-\text{x}\right)}^{\frac{1}{\text{P}}}\right)$. Note that, if separately integrating the exponential function and the functional form implied by Pareto distribution from 0 to 1, it can be seen that both functional forms have the same area under the Lorenz curve which is equal to $\frac{1}{\text{P}+1}$. By assigning the weight (1−k) to the exponential function and the weight k to the functional form implied by Pareto distribution, the specified functional form based on the weighted average of these 2 well-known functional forms is as follows:

(1) $$\text{y}\left(\text{x}\right)=\left[1-\text{k}\right]\text{∗}{\text{x}}^{\text{P}}+\text{k∗}\left[1-{\left(1-\text{x}\right)}^{\frac{1}{\text{P}}}\right]$$

0≤k≤1

1≤P

**Figure 1:**
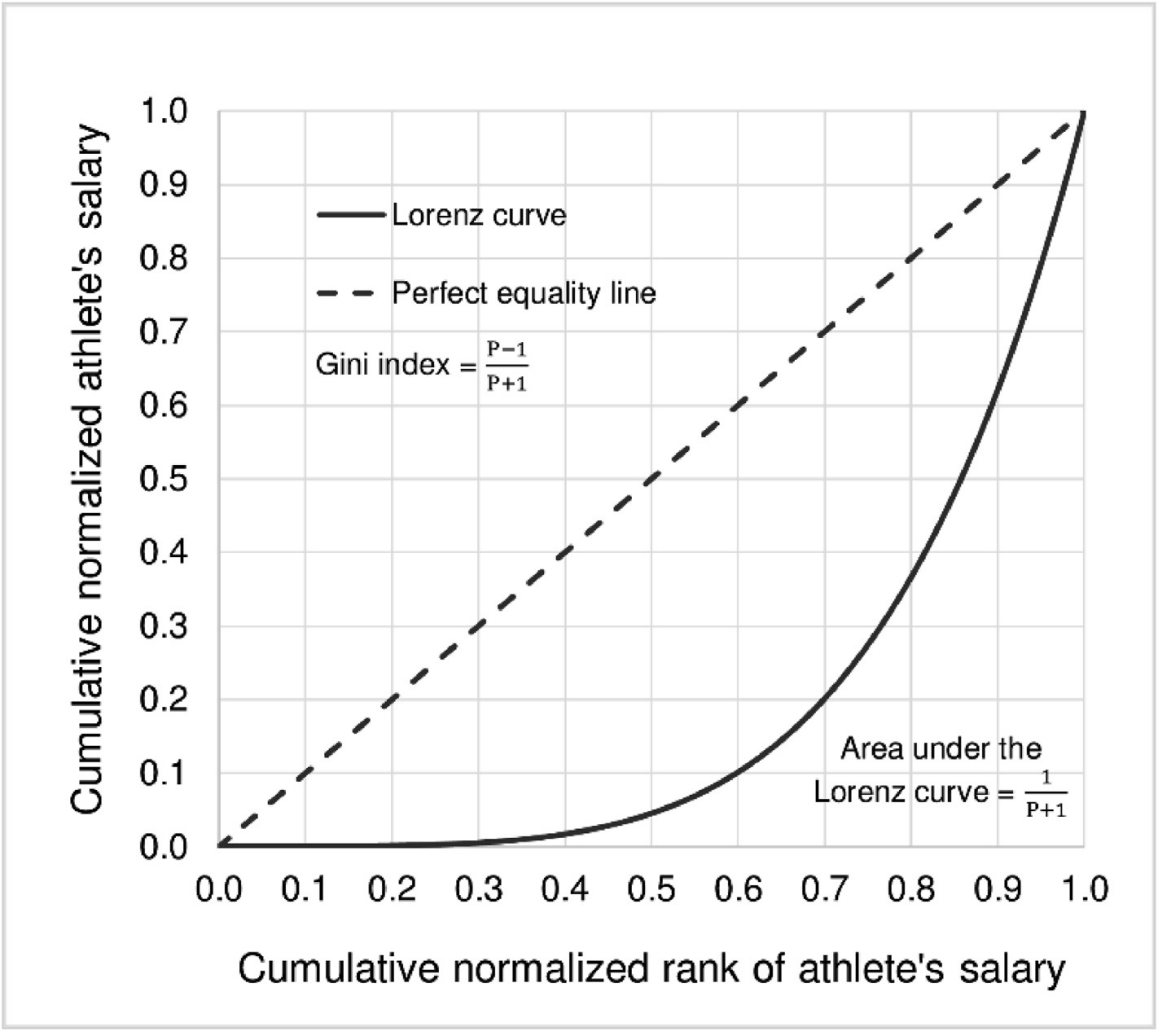
The Lorenz curve and the Gini index.

This specified functional form satisfies all required properties of the Lorenz curve in that it must be a monotonically increasing function $\left(\frac{\text{dy}}{\text{dx}}\geq 0,\frac{{\text{d}}^{2}\text{y}}{{\text{dx}}^{2}}\geq 0\right)$ and must pass two coordinates which are (0, 0) and (1, 1) as illustrated in Figure 1.

According to Sitthiyot and Holasut (2021), the parameter k is the weight that controls the curvature of the estimated Lorenz curve such that the Gini index remains constant, whereas the parameter P represents the degree of inequality in the distribution of the athlete’s salary. The values of parameters k and P can vary depending upon the curvature of the estimated Lorenz curve and the degree of inequality in the distribution of the athlete’s salary for each professional sport. The estimated Lorenz curve based on Eq. (1) would be used to calculate the Gini index for the athlete’s salary which measures inequality in the distribution of salary of the athlete for each type of 11 professional sports. The Gini index takes values between 0 and 1. The closer the index is to 0, the more equal the distribution of the athlete’s salary. The closer the index is to 1, the more unequal the distribution of the athlete’s salary. Sitthiyot and Holasut (2020) note that the advantage of the Gini index as a measure of income inequality is that the inequality of the entire income distribution could be summarized by using a single number that is easy to interpret since its values are bounded between 0 and 1. This would allow for comparison among countries with different population sizes. Moreover, the data on the Gini index is easy to access, regularly updated and published by countries and/or international organizations. For these reasons, the Gini index has arguably been the most popular measure of inequality despite the fact that there are well over 50 inequality indices as discussed in Coulter (1989).

Given the estimated Lorenz curve for each of 11 professional sports, we can calculate the area under the curve by integrating the estimated Lorenz equation from 0 to 1 $\left(\overset{1}{\underset{0}{\int }}\text{y}\left(\text{x}\right)\text{dx}\right)$. Based on the parametric functional form proposed by Sitthiyot and Holasut (2021) as shown in Eq. (1), the area under the curve equals $\frac{1}{\text{P}+1}$. After obtaining the area under the estimated Lorenz curve, the Gini index for the athlete’s salary for each type of 11 professional sports can be calculated as $1-2\text{∗}\overset{1}{\underset{0}{\int }}\text{y}\left(\text{x}\right)\text{dx}$ which is equal to $\frac{\text{P}-1}{\text{P}+1}$. Note that when P=1, the Gini index =0, which implies perfect equality (all athletes in the same professional sports receive the same salary share). When P=∞, the Gini index =1, which implies perfect inequality (one athlete has all the salary and the rest has none for the same professional sports).

In addition to the Gini index for the athlete’s salary, the estimated Lorenz equation can be used to compute the salary shares by quintile for each of 11 professional sports. Thus, each of 11 professional sports would have its own Gini index and the salary shares by quintile where the 1^st^ quintile is the salary share held by the lowest 20% (0% ≤ salary share ≤ 20%), the 2^nd^ quintile is the salary share held by the second 20% (20% < salary share ≤ 40%), the 3^rd^ quintile is the salary share held by the third 20% (40% < salary share ≤ 60%), the 4^th^ quintile is the salary share held by the fourth 20% (60% < salary share ≤ 80%), and the 5^th^ quintile is the salary share held by the top 20% (80% < salary share ≤ 100%).

Provided that the distributions of the athlete’s salary of all 11 professional sports satisfy the notions of fairness according to the concepts of procedural justice and distributive justice together with the concept of power of authority which reflect the no-envy principle of fair allocation introduced by Tinbergen (1930) and the general consensus or the international norm criterion for a meaningful benchmark according to the OHCHR (2012) as discussed in Introduction, the next step is to construct the fairness lines. This could be done by estimating the statistical relationship between the salary shares of the athlete in each quintile (Q^S^_i_, i = 1, 2, …, 5) and the Gini index for the athlete’s salary (${\text{Gini}}_{\text{S}}$) using the data from each type of 11 professional sports. Note that, for both theoretical and practical purposes with regard to the mathematical properties of the Lorenz curve and those of the Gini index, we impose five conditions when estimating the relationship between the salary shares of the athlete in each quintile (Q^S^_i_, i = 1, 2, …, 5) and the Gini index for the athlete’s salary (${\text{Gini}}_{\text{S}}$). The first condition is that the fairness line for the 5^th^ quintile must pass 2 coordinates which are (0, 0.2) and (1, 1). The justification for the first condition is that when the Gini index for the athlete’s salary is equal to 0, the salary share in the 5^th^ quintile must be equal to 0.2, and when the Gini index for the athlete’s salary is equal to 1, the salary share in the 5^th^ quintile must be equal to 1. For the second condition, the fairness line for the other 4 quintiles must pass 2 coordinates which are (0, 0.2) and (1, 0). The rationale for the second condition is that when the Gini index for the athlete’s salary equals 0, the salary shares in the 1^st^, the 2^nd^, the 3^rd^, and the 4^th^ quintile all have to be equal to 0.2. When the Gini index for the athlete’s salary is equal to 1, the salary shares in the 1^st^, the 2^nd^, the 3^rd^, and the 4^th^ quintile all have to be equal to 0. Given that the property of the Lorenz curve must be a monotonically increasing function, the third condition is that, for a given value of the Gini index for the athlete’s salary, 0 ≤ the salary share in the 1^st^ quintile (Q^S^_1_) ≤ the salary share in the 2^nd^ quintile (Q^S^_2_) ≤ the salary share in the 3^rd^ quintile (Q^S^_3_) ≤ the salary share in the 4^th^ quintile (Q^S^_4_) ≤ the salary share in the 5^th^ quintile (Q^S^_5_) ≤ 1. For the fourth condition, ${\frac{{\text{dQ}}_{1}^{\text{s}}}{{\text{dGini}}_{\text{s}}}|}_{{\text{Gini}}_{\text{S}}=0}\leq \;{\frac{{\text{dQ}}_{2}^{\text{s}}}{{\text{dGini}}_{\text{s}}}|}_{{\text{Gini}}_{\text{S}}=0}\leq \;{\frac{{\text{dQ}}_{3}^{\text{s}}}{{\text{dGini}}_{\text{s}}}|}_{{\text{Gini}}_{\text{S}}=0}\leq \;{\frac{{\text{dQ}}_{4}^{\text{s}}}{{\text{dGini}}_{\text{s}}}|}_{{\text{Gini}}_{\text{S}}=0}\leq \;{\frac{{\text{dQ}}_{5}^{\text{s}}}{{\text{dGini}}_{\text{s}}}|}_{{\text{Gini}}_{\text{S}}=0}$. That is when the Gini index for the athlete’s salary = 0, the slope of the estimated fairness line for the 1^st^ quintile ≤ the slope of the estimated fairness line for the 2^nd^ quintile ≤ the slope of the estimated fairness line for the 3^rd^ quintile ≤ the slope of the estimated fairness line for the 4^th^ quintile ≤ the slope of the estimated fairness line for the 5^th^ quintile. Regarding the fifth condition, the summation of the salary shares by quintile $\left(\overset{5}{\underset{\text{i}=1}{\sum }}{\text{Q}}_{\text{i}}^{\text{S}}\right)$ for each professional sport must equal 1, for a given value of the Gini index for the athlete’s salary. Given these five conditions, the curve fitting technique based on minimizing error sum of squares is applied in order to find the statistical relationship between the salary shares of the athlete in each quintile (Q^S^_i_, i = 1, 2, …, 5) and the Gini index for the athlete’s salary (${\text{Gini}}_{\text{S}}$). This would give us 5 fairness lines, one for each quintile, that could be used to estimate the fair salary shares in each quintile for a given value of the Gini index for the athlete’s salary.

To measure whether the income distribution of a country is fair or not, we plot the Cartesian coordinate where the abscissa is the income Gini index and the ordinate is the income share in each quintile of that country (Q^I^_i_, i = 1, 2, …, 5). We then compare to see whether the ordered-pair (income Gini index, Q^I^_i_) is on, above, or below the fairness line for the same value of the income Gini index. The closer the ordered-pair (income Gini index, Q^I^_i_) of a country is to the fairness line, the fairer the income share in that quintile relative to the professional athlete’s salary benchmark. If the ordered-pair (income Gini index, Q^I^_i_) of a country lies above the fairness line, it indicates that the income earners in that quintile receive income share more than the fair share. In contrast, if the ordered-pair (income Gini index, Q^I^_i_) of a country lies below the fairness line, it suggests that the income earners in that quintile receive income share lower than the fair share. In practice, for an existing value of the income Gini index of a country, the degree of fairness in income shares by quintile could be quantitatively measured by calculating the percentage deviation (in absolute value) of a country’s actual income shares in each quintile from the corresponding fair quintile income shares based on the professional athlete’s salary benchmark. In this way, not only would our method be able to identify which quintile the income share of a country is fair or not fair relative to the benchmark derived from the athlete’s salary from 11 professional sports, but also suggest what the fair share of income in each quintile of that country should be for an existing value of the Gini index. For a comparison of fair income distributions across countries that have the same Gini index, this could be done by taking the sum of the percentage deviation (in absolute value) of each country’s actual quintile income shares from the corresponding fair quintile income shares consistent with the professional athlete’s salary benchmark. A country that has a lower figure of the sum of the percentage deviation (in absolute value) of the actual quintile income shares from the corresponding fair quintile income shares would have a fairer income distribution than the other countries.

In this study, the data on the annual salaries of the athletes from 11 professional sports in 2019 or the latest from Sitthiyot (2021) comprising 6,709 observations are used in order to construct the fairness lines. The data on the income Gini index and the income shares by quintile in 2015 from the World Bank (2020a) comprising 75 countries are employed in order to demonstrate how the fairness lines could be used as a benchmark for quantitatively measuring whether the income distribution of a country is fair or not. Note that the reason we use the data on the income Gini index and the income shares by quintile in 2015 for demonstrating our method is mainly because there are more observations than those in 2016 and 2017, and no data on the Gini index and income shares by quintile were available for 2018 and 2019 at the time we accessed the World Bank database. Table 1 reports the descriptive statistics of the athlete’s salary from 11 professional sports while the descriptive statistics of the income Gini index and the income shares by quintile are provided in Table 2.

**Table 1:** The descriptive statistics of the athlete’s salary from 11 professional sports. The unit of currency is the United States dollar except for the EPL where the unit of currency is the Pound Sterling.

Type of sports	Mean	Median	Mode	Minimum	Maximum	Standard deviation	No. of players
1. WNBA	73,190.60	59,718	115,000	2,723	127,500	32,589.64	151
2. EPL	2,695,133.83	1,976,000	1,820,000	0.00	19,500,000	2,489,890.55	523
3. NFL	4,682,534.29	3,000,000	3,095,000	831,349	30,700,000	4,525,065.24	1,000
4. NHL	2,618,049.97	1,237,500	700,000	675,000	12,500,000	2,396,904.17	1,000
5. MLB	7,985,791.19	5,000,000	4,000,000	583,500	37,666,666	7,708,701.88	481
6. NBA	7,600,037.45	3,500,000	1,620,564	208,509	40,231,758	8,767,208.31	517
7. PGA	1,235,495.42	838,030	-	5,910	9,684,006	1,433,077.03	264
8. LPGA	39,064.90	21,380	10,874	4,015	313,272	58,022.09	77
9. MLS	409,288.28	175,135	70,250	56,250	7,200,000	718,621.46	658
10. ATP	37,474.00	1,084	54	54	3,915,011	158,294.82	1,070
11. WTA	27,520.67	625	147	37	2,916,508	122,838.31	968

**Table 2:** The descriptive statistics of the income Gini index and the income shares by quintile (in decimals) of 75 countries.

Indicator	Mean	Median	Mode	Minimum	Maximum	Standard deviation	No. of countries
Income Gini index	0.369	0.356	0.318	0.254	0.591	0.081	75
Income share held by the top 20%	0.441	0.421	0.384	0.350	0.637	0.066	75
Income share held by the fourth 20%	0.219	0.221	0.226	0.179	0.247	0.012	75
Income share held by the third 20%	0.158	0.163	0.175	0.098	0.187	0.019	75
Income share held by the second 20%	0.115	0.118	0.121	0.058	0.147	0.021	75
Income share held by the lowest 20%	0.068	0.071	0.078	0.028	0.100	0.019	75

## 3 Results

We first report the values of parameters k and P as well as the values of coefficient of determination (R^2^) for the estimated Lorenz equations for each type of 11 professional sports which are WNBA, EPL, NFL, NHL, MLB, NBA, PGA, LPGA, MLS, ATP, and WTA. The values of k and P are estimated by using Eq. (1) and the curve fitting technique based on minimizing error sum of squares as described in Materials and Methods. They are shown in Table 3.

**Table 3:** The values of parameters k and P as well as R^2^ from the estimated Lorenz curves for 11 types of professional sports.

Type of sports	k	P	R^2^
1. WNBA	0.21	1.66	0.9976
2. EPL	0.39	2.62	0.9995
3. NFL	0.50	2.76	0.9999
4. NHL	0.48	2.76	0.9990
5. MLB	0.28	2.96	0.9986
6. NB	0.31	3.52	0.9949
7. PGA	0.25	3.55	0.9988
8. LPGA	0.48	4.04	0.9951
9. MLS	0.55	4.05	0.9999
10. ATP	0.09	14.39	0.9999
11. WTA	0.08	14.69	0.9999

Note that the estimated Lorenz equations fit the actual Lorenz plots that represent the relationship between the cumulative normalized athlete’s salary on the vertical axis and the cumulative normalized rank of the athlete’s salary on the horizontal axis for each of 11 professional sports quite well with the values of R^2^ ranging between 0.9949 and 0.9999.

We then use these estimated Lorenz curves to calculate the values of the Gini index for the athlete’s salary (${\text{Gini}}_{\text{S}}$) and the salary shares by quintile (Q^S^_i_, i = 1, 2, …, 5) (in decimals) for each of 11 professional sports. The results are reported in Table 4.

**Table 4:** The values of the Gini index for the athlete’ salary and the salary shares by quintile (in decimals) for each type of the 11 professional sports. The 2 conditions are also included which are perfect equality and perfect inequality where the Gini index for the athlete’s salary takes the values of 0 and 1, respectively.

Type of sports	$Gini_{S}$	Q^S^_5_	Q^S^_4_	Q^S^_3_	Q^S^_2_	Q^S^_1_
Perfect equality	0.000	0.200	0.200	0.200	0.200	0.200
1. WNBA	0.247	0.323	0.248	0.199	0.148	0.081
2. EPL	0.447	0.480	0.244	0.151	0.084	0.041
3. NFL	0.468	0.509	0.228	0.139	0.080	0.045
4. NHL	0.469	0.507	0.231	0.140	0.079	0.043
5. MLB	0.495	0.511	0.256	0.141	0.066	0.026
6. NBA	0.557	0.571	0.243	0.116	0.048	0.021
7. PGA	0.560	0.569	0.252	0.117	0.045	0.018
8. LPGA	0.603	0.631	0.205	0.094	0.043	0.027
9. MLS	0.604	0.637	0.195	0.092	0.046	0.030
10. ATP	0.870	0.954	0.040	0.003	0.002	0.001
11. WTA	0.873	0.957	0.038	0.003	0.002	0.001
Perfect inequality	1.000	1.000	0.000	0.000	0.000	0.000

Excluding the cases of perfect equality and perfect inequality, the results in Table 4 indicate that the degree of inequality in the distributions of athlete’s salary, measured by the Gini index, could be as low as 0.247 (WNBA) and as high as 0.873 (WTA). In addition, the salary share of the top 20% (Q^S^_5_) could be as low as 0.323 (WNBA) and as high as 0.957 (WTA) whereas the salary share of the bottom 20% (Q^S^_1_) could be as low as 0.001 (ATP and WTA) and as high as 0.081(WNBA), resulting in the salary gap between the top 20% (Q^S^_5_) and the bottom 20% (Q^S^_1_) to be as low as 3.99 times (WNBA) and as high as 957 times (WTA). These results clearly illustrate that a high degree of inequality in the distribution of salary and a substantial salary gap between the top 20% and the bottom 20% could be acceptable if they are procedurally and distributively viewed as fair. Table 5 reports the descriptive statistics of the Gini index for the athlete’s salary and the salary shares by quintile of 11 professional sports.

**Table 5:** The descriptive statistics of the Gini index for the athlete’s salary and the salary shares by quintile (in decimals) of 11 professional sports. The salary shares when the Gini index = 0.000 and 1.000 are excluded when computing the descriptive statistics.

Indicator	Mean	Median	Mode	Minimum	Maximum	Standard deviation	No. of sports
Gini index for athletes' salaries	0.5630	0.5575	-	0.2470	0.8725	0.1815	11
Salary share held by the top 20%	0.6045	0.5690	-	0.3233	0.9568	0.1930	11
Salary share held by the fourth 20%	0.1982	0.2310	-	0.0377	0.2562	0.0811	11
Salary share held by the third 20%	0.1086	0.1168	-	0.0027	0.1995	0.0600	11
Salary share held by the second 20%	0.0582	0.0478	-	0.0016	0.1476	0.0408	11
Salary share held by the lowest 20%	0.0304	0.0266	-	0.0013	0.0813	0.0224	11

**Figure 2:**
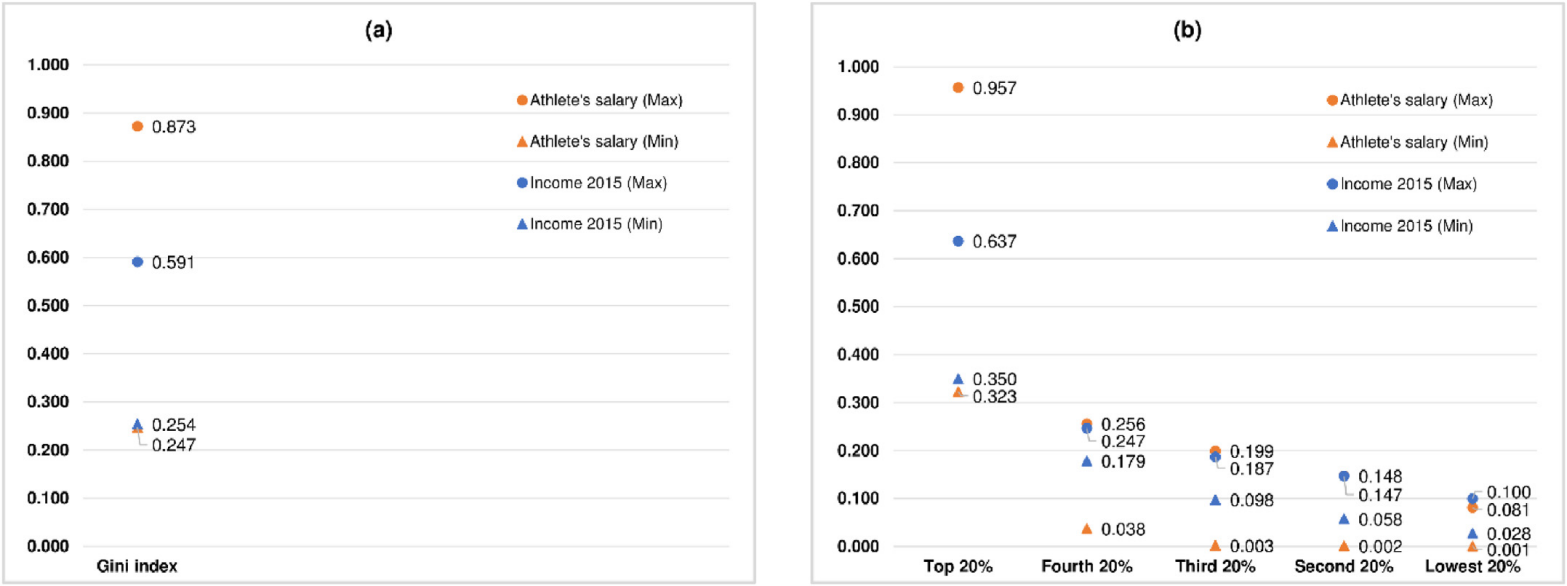
The minimum and the maximum values of the Gini index for the athlete’s salary and those of the salary shares by quintile vs. the minimum and the maximum values of the income Gini index and those of the income shares by quintile in 2015. (a) Gini index. (b) Income shares by quintile.

The descriptive statistics of the Gini index for the athlete’s salary and the salary shares by quintile as reported in Table 5 could be compared to the descriptive statistics of the income Gini index and the income shares by quintile in 2015 as shown in Table 2. Figure 2 shows the plots of the minimum and the maximum values of the Gini index for the athlete’s salary and those of the salary shares by quintile vs. the plots of the minimum and the maximum values of the income Gini index and those of the income shares by quintile in 2015.

It can be seen from Figures 2a and 2b that the minimum and the maximum values of the income Gini index and the income shares by quintile in 2015 lie within the corresponding minimum and maximum values of the Gini index for the athlete’s salary and the salary shares by quintile except for the lowest 20% where the maximum value of the income share (0.100) is slightly higher than that of the salary share (0.081). In addition, we collect the data on the income Gini index and the income shares by quintile for the entire period between 1995 and 2015 from the World Bank (2020a) and find their minimum and maximum values. The findings indicate that the minimum and the maximum values of the income Gini index and the income shares by quintile from 1995 to 2015, by and large, lie within the corresponding minimum and maximum values of the Gini index for the athlete’s salary and the salary shares by quintile. Out of 252 observations on the minimum and the maximum values of the income Gini index and the income shares by quintile from 1995 to 2015, there are 9 observations on the income Gini index whose values (0.230–0.246) are slightly below the minimum value of the Gini index for the athlete’s salary (0.247) while there are 21 observations on the income share of the lowest 20% whose values (0.090–0.109) are just above the maximum value of the salary share of the lowest 20% (0.081). This is to show that, for the values of the Gini index for the athlete’s salary and those of the salary shares by quintile to be used for developing the fairness benchmark, they should, by and large, cover not only the values of the income Gini index and those of the income shares by quintile of 75 countries in the sample but also the values of the income Gini index and those of the income shares by quintile across a wide range of countries and time periods.

Given the allocations of the athlete’s salary of all 11 professional sports are resulted from fair and transparent rules written and administered by international sports authorities, individual and/or team performance, as well as luck, all of which correspond to the notions of procedural justice, distributive justice, and authority’s power as discussed in Introduction, the values of the Gini index for the athlete’s salary (${\text{Gini}}_{\text{S}}$) and the salary shares by quintile (Q^S^_i_, i = 1, 2, …, 5) can thus be used to construct the fairness lines, one for each quintile. This could be done by estimating the statistical relationship between the salary shares of the athlete in each quintile and the Gini index for the athlete’s salary based on five conditions which are: 1) the fairness line for the 5^th^ quintile has to pass 2 coordinates, namely (0, 0.2) and (1, 1); 2) the fairness lines for the other 4 quintiles must pass 2 coordinates, namely (0, 0.2) and (1, 0); 3) for a given value of the Gini index for the athlete’s salary, 0 ≤ the salary share in the 1^st^ quintile (Q^S^_1_) ≤ the salary share in the 2^nd^ quintile (Q^S^_2_) ≤ the salary share in the 3^rd^ quintile (Q^S^_3_) ≤ the salary share in the 4^th^ quintile (Q^S^_4_) ≤ the salary share in the 5^th^ quintile (Q^S^_5_) ≤ 1; 4) ${\frac{{\text{dQ}}_{1}^{\text{s}}}{{\text{dGini}}_{\text{s}}}|}_{{\text{Gini}}_{\text{S}}=0}\leq \;{\frac{{\text{dQ}}_{2}^{\text{s}}}{{\text{dGini}}_{\text{s}}}|}_{{\text{Gini}}_{\text{S}}=0}\leq \;{\frac{{\text{dQ}}_{3}^{\text{s}}}{{\text{dGini}}_{\text{s}}}|}_{{\text{Gini}}_{\text{S}}=0}\leq \;{\frac{{\text{dQ}}_{4}^{\text{s}}}{{\text{dGini}}_{\text{s}}}|}_{{\text{Gini}}_{\text{S}}=0}\leq \;{\frac{{\text{dQ}}_{5}^{\text{s}}}{{\text{dGini}}_{\text{s}}}|}_{{\text{Gini}}_{\text{S}}=0}$; and 5) for a given value of the Gini index for the athlete’s salary, the summation of the salary shares by quintile $\left(\overset{5}{\underset{\text{i}=1}{\sum }}{\text{Q}}_{\text{i}}^{\text{S}}\right)$ for each sports must be equal to 1. These five conditions are imposed in order to guarantee that the estimated fairness lines satisfy the mathematical properties of the Lorenz curve and those of the Gini index. By applying the curve fitting technique based on minimizing sum of squared errors, we find that the estimated equations for the fairness lines can be fitted by the 4^th^ degree polynomial function. They are as follows:

(2) $${\text{Q}}_{1}^{\text{S}}=0.1956{\text{Gini}}_{\text{S}}^{4}-0.6612{\text{Gini}}_{\text{S}}^{3}+0.9356{\text{Gini}}_{\text{S}}^{2}-0.6700{\text{Gini}}_{\text{S}}+0.20,{\text{R}}^{2}=0.9836$$

(3) $${\text{Q}}_{2}^{\text{S}}=-0.4914{\text{Gini}}_{\text{S}}^{4}+1.3968{\text{Gini}}_{\text{S}}^{3}-1.1195{\text{Gini}}_{\text{S}}^{2}+0.0141{\text{Gini}}_{\text{S}}+0.20,{\text{R}}^{2}=0.9935$$

(4) $${\text{Q}}_{3}^{\text{S}}=0.2545{\text{Gini}}_{\text{S}}^{4}+0.0508{\text{Gini}}_{\text{S}}^{3}-0.6650{\text{Gini}}_{\text{S}}^{2}+0.1597{\text{Gini}}_{\text{S}}+0.20,{\text{R}}^{2}=0.9915$$

(5) $${\text{Q}}_{4}^{\text{S}}=1.9481{\text{Gini}}_{\text{S}}^{4}-3.3533{\text{Gini}}_{\text{S}}^{3}+1.0625{\text{Gini}}_{\text{S}}^{2}+0.1428{\text{Gini}}_{\text{S}}+0.20,{\text{R}}^{2}=0.9737$$

(6) $${\text{Q}}_{5}^{\text{S}}=-1.9067{\text{Gini}}_{\text{S}}^{4}+2.5669{\text{Gini}}_{\text{S}}^{3}-0.2136{\text{Gini}}_{\text{S}}^{2}+0.3534{\text{Gini}}_{\text{S}}+0.20,{\text{R}}^{2}=0.9960$$

**Figure 3:**
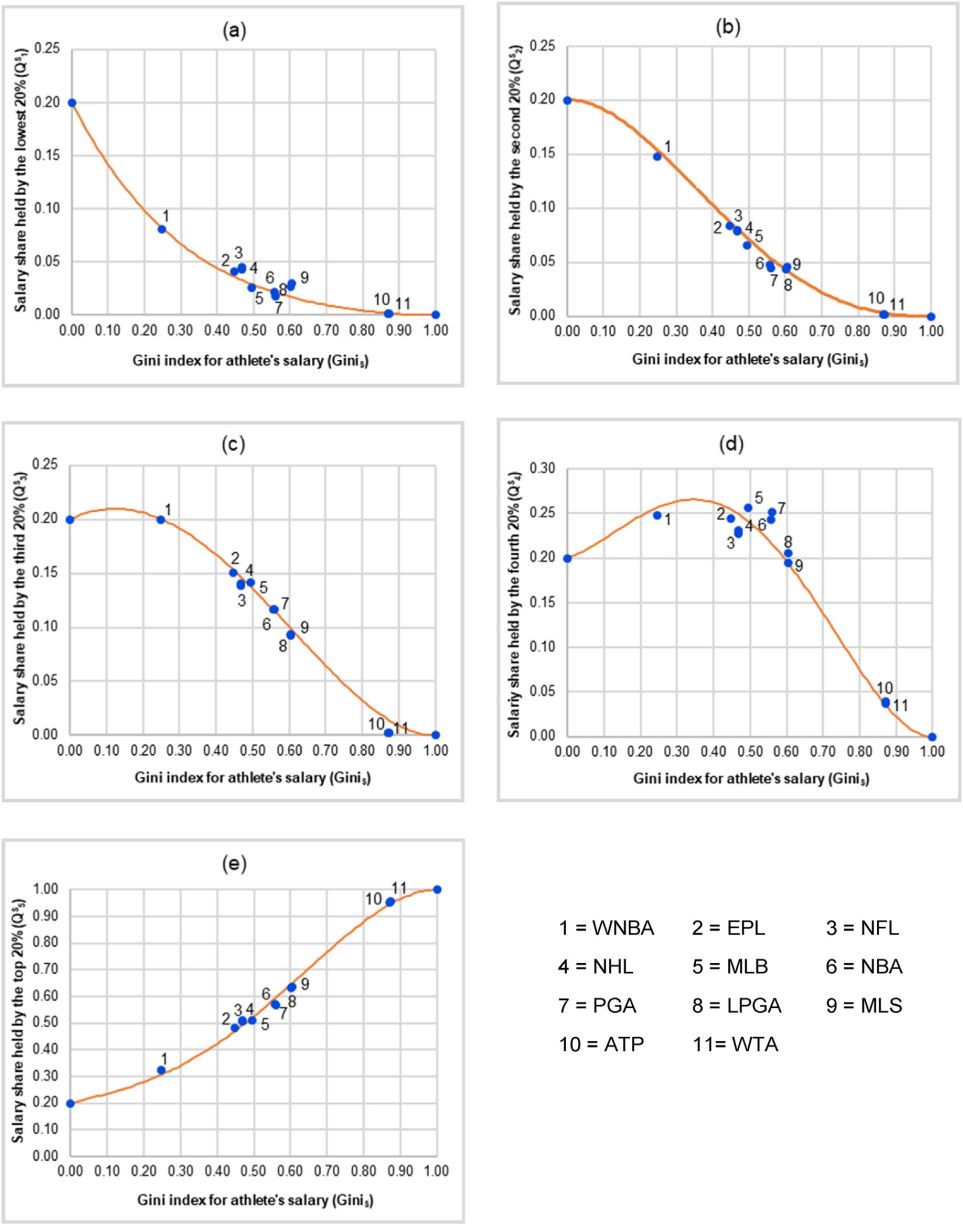
The estimated fairness lines representing the relationship between the salary shares in each quintile (Q^S^_i_, i = 1, 2, …, 5) and the Gini index for the athlete’s salary ($Gini_{S}$) from 11 professional sports. (a) Salary share held by the lowest 20% (Q^S^_1_). (b) Salary share held by the second 20% (Q^S^_2_). (c) Salary share held by the third 20% (Q^S^_3_). (d) Salary share held by the fourth 20% (Q^S^_4_). (e) Salary share held by the top 20% (Q^S^_5_).

Figure 3 illustrates the fairness lines representing the relationship between the salary shares of the professional athlete in each quintile and the Gini index for the athlete’s salary.

Next, we show how the fairness lines could be used as a benchmark for measuring whether income shares in each quintile of a country are regarded as fair or not. This could be done by comparing the Cartesian coordinates of each country in the sample where the abscissa is the income Gini index and the ordinate is the income shares by quintile (Q^I^_i_, i = 1, 2, …, 5) to the fairness lines in the corresponding quintiles as shown in Figure 3. The closer the ordered-pair (income Gini index, Q^I^_i_) of a country is to the fairness line, the fairer the income share in that quintile. The further away the ordered-pair (income Gini index, Q^I^_i_) is from the fairness line, the more unfair the income share of a country in that quintile. In this study, we measure the degree of fairness by calculating the percentage deviation (in absolute value) of a country’s income share in each quintile from the fair income share based on our benchmark derived from the relationship between the salary shares of the athlete in each quintile and the Gini index for the athlete’s salary from 11 professional sports.

**Figure 4:**
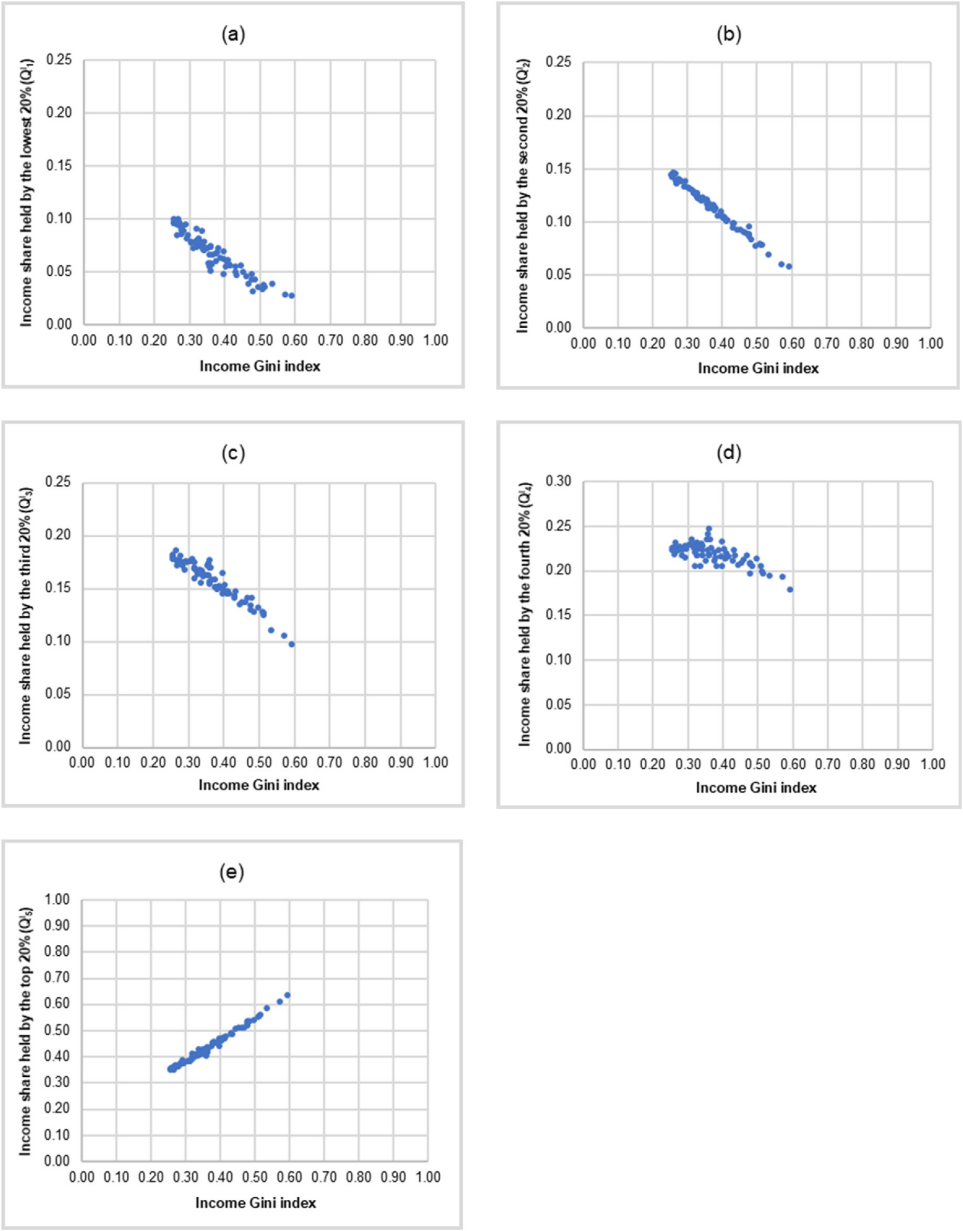
Scatter plots illustrating the relationship between the income Gini index and the income shares by quintile (Q^I^_i_, i = 1, 2, …, 5) of 75 countries. (a) Income share held by the lowest 20% (Q^I^_1_). (b) Income share held by the second 20% (Q^I^_2_). (c) Income share held by the third 20% (Q^I^_3_). (d) Income share held by the fourth 20% (Q^I^_4_). (e) Income share held by the top 20% (Q^I^_5_).

Using the data on the income Gini index and the income shares by quintile in 2015 (in decimals) from the World Bank (2020a) covering 75 countries, Figure 4 illustrates the scatter plots of the Cartesian coordinate where the abscissa is the income Gini index and the ordinate is the income shares by quintile of 75 countries in the sample (Q^I^_i_, i = 1, 2, …, 5).

**Figure 5:**
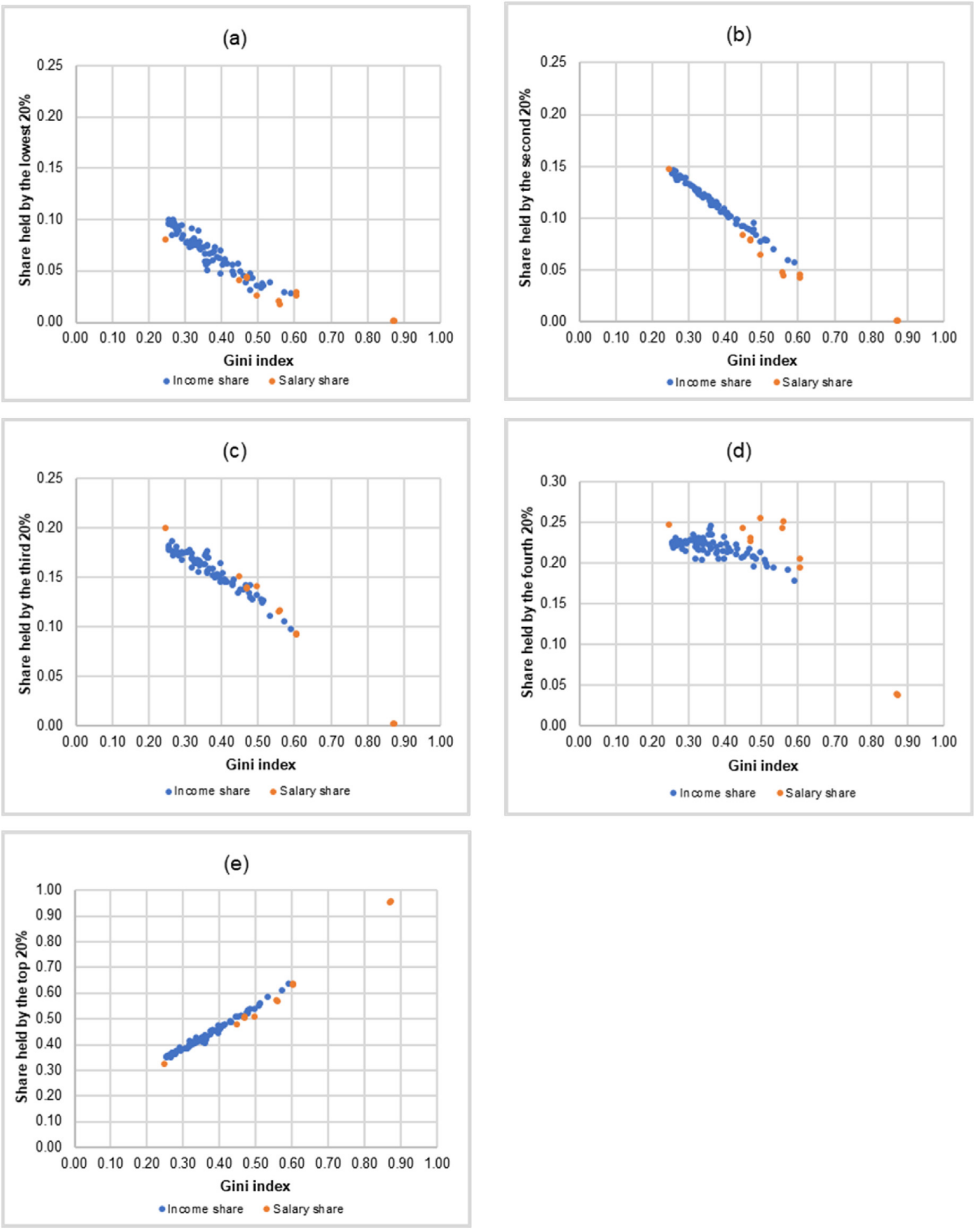
Similarity between the scatter plots of the ordered-pairs of the income Gini index and the income shares by quintile (Income Gini index, Q^I^_i_) of 75 countries and the scatter plots of the ordered-pairs of the Gini index for the athlete’s salary and the salary shares by quintile (Gini_S_, Q^S^_i_) of 11 professional sports. (a) Share held by the lowest 20%. (b) Share held by the second 20%. (c) Share held by the third 20%. (d) Share held by the fourth 20%. (e) Share held by the top 20%.

It is interesting to note that the scatter plots of the ordered-pairs (income Gini index, Q^I^_i_) of all 75 countries in the corresponding quintile as shown in Figure 4 share similar pattern with the scatter plots of the ordered-pairs (${\text{Gini}}_{\text{S}}$, Q^S^_i_) of 11 professional sports in each quintile used to construct the fairness lines as shown in Figure 3. The scatter plots of the ordered-pairs (${\text{Gini}}_{\text{S}}$, Q^S^_i_) of 11 professional sports in Figure 3 and the scatter plots of the ordered-pairs (income Gini index, Q^I^_i_) of 75 countries in Figure 4 could be viewed together which are shown in Figure 5.

**Figure 6:**
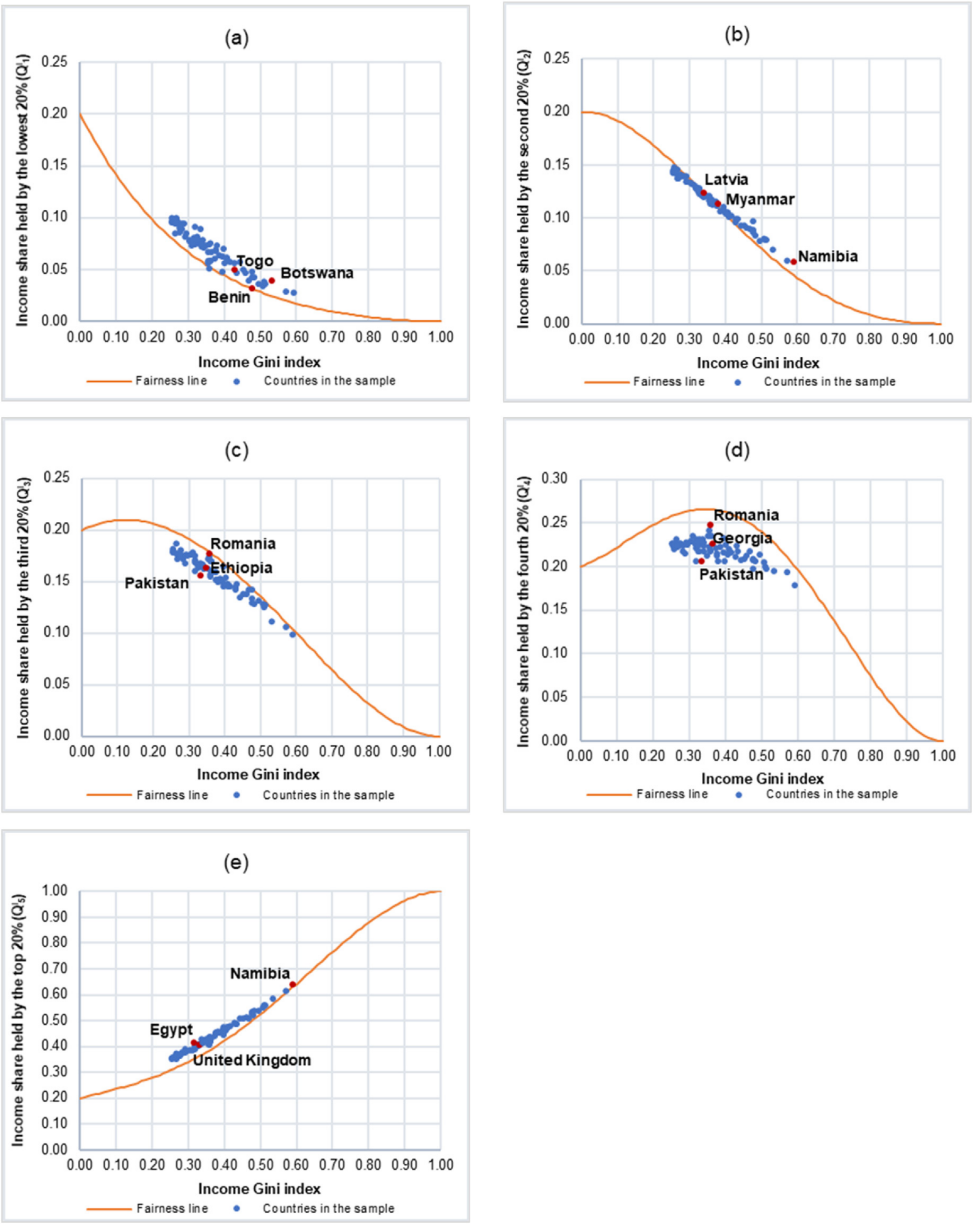
Scatter plots of the Cartesian coordinates of the income Gini index and the income shares by quintile (Q^I^_i_, i = 1, 2, …, 5) of 75 countries. The fairness lines are used as a benchmark for analyzing whether the income share of each of 75 countries in each quintile is a fair share or not for a particular value of the income Gini index of that country. (a) Income share held by the lowest 20% (Q^I^_1_). (b) Income share held by the second 20% (Q^I^_2_). (c) Income share held by the third 20% (Q^I^_3_). (d) Income share held by the fourth 20% (Q^I^_4_). (e) Income share held by the top 20% (Q^I^_5_).

Figure 6 illustrates how the fairness lines could be used to quantitatively analyze fair income distribution by comparing the scatter plots of the Cartesian coordinates of 75 countries in the sample where the abscissa is the income Gini index and the ordinate is the income shares by quintile (Q^I^_i_, i = 1, 2, …, 5) to the corresponding fairness lines in each quintile.

The overall results shown in Figure 6 indicate that the ordered-pairs (income Gini index, Q^I^_i_) of all 75 countries lie either above or below the fairness lines in all 5 quintiles, with those in the 1^st^ and the 5^th^ quintile being above their corresponding fairness lines (except Romania whose point is just below the fairness line in the 1^st^ quintile) and those in the 3^rd^ and the 4^th^ quintile being below their corresponding fairness lines. For the 2^nd^ quintile, there are 35 countries whose ordered-pairs (income Gini index, Q^I^_i_) lie above the fairness line while there are 40 countries whose ordered-pairs (income Gini index, Q^I^_i_) lie below the fairness line. The results from the sample of 75 countries suggest that, relative to our professional athlete’s salary benchmark, the income earners in the 1^st^ (except Romania), the 2^nd^ (35 countries), and the 5^th^ quintile receive income shares higher than the fair shares, with those in the 1^st^ quintile receive the highest share as measured by the absolute values of the maximum (60.19%), the mean (29.98%), and the median (28.75%) of country’s income share, whereas the income earners in the 2^nd^ (40 countries), the 3^rd^, and the 4^th^ quintile receive income shares lower than the fair shares, with those in the 4^th^ quintile receive the lowest share as measured by the absolute values of the minimum (22.80%), the mean (15.11%), and the median (14.78%) of country’s income share.

The results from each quintile, starting from the 1^st^ quintile where the income share is held by the lowest 20% (Figure 6a), show that, for an existing value of a country’s Gini index, the income earners in 74 out of 75 countries have income shares higher than the fair shares as suggested by our professional athlete’s salary benchmark. The country whose income share deviates from the fair share the most is Botswana (60.19%) while that of Benin deviates the least (1.58%), with the median country being Togo (28.75%). For the 2^nd^ quintile where the income share is held by the second 20% (Figure 6b), Namibia is regarded as having the most unfair income share since the income earners in Namibia receive 26.97% higher than the fair share according to our professional athlete’s salary benchmark, whereas the least unfair income share country is Latvia, with merely 0.02% lower than the fair share. The median country in the 2^nd^ quintile is Myanmar whose income share is higher than the fair share by 2.96%. The results from the 3^rd^ quintile where the income share is held by the third 20% (Figure 6c) show that, compared to the professional athlete’s salary benchmark, the income earners in Pakistan receive income share lower than the fair share the most (15.22%) while those in Romania receive income share lower than the fair share the least (0.68%), with Ethiopia being the median country whose income earners receive 9.67% lower than the fair share. For the 4^th^ quintile where the income share is held by the fourth 20% (Figure 6d), our results show that the income earners in Pakistan receive income share lower than the fair share the most as indicated by our professional athlete’s salary benchmark (22.80%). The country whose income earners receive income share lower than the fair share the least is Romania (6.93%). Georgia is the median country in the 4^th^ quintile whose income earners receive income share lower than the fair share by 14.78%. Lastly, our results for the 5^th^ quintile where the income share is held by the top 20% (Figure 6e) indicate that, relative to our professional athlete’s salary benchmark, the country whose income earners receive income share higher than the fair share the most is Egypt (17.29%) while the income earners in Namibia receive income share higher than the fair share the least (0.87%), with the United Kingdom being the median country whose income earners receive income share higher than the fair share by 11.37%. The actual income shares by quintile vs. the fair income shares by quintile as well as the values of the Gini index for all 75 countries in the sample are reported in Table A1 in Appendix.

**Figure 7:**
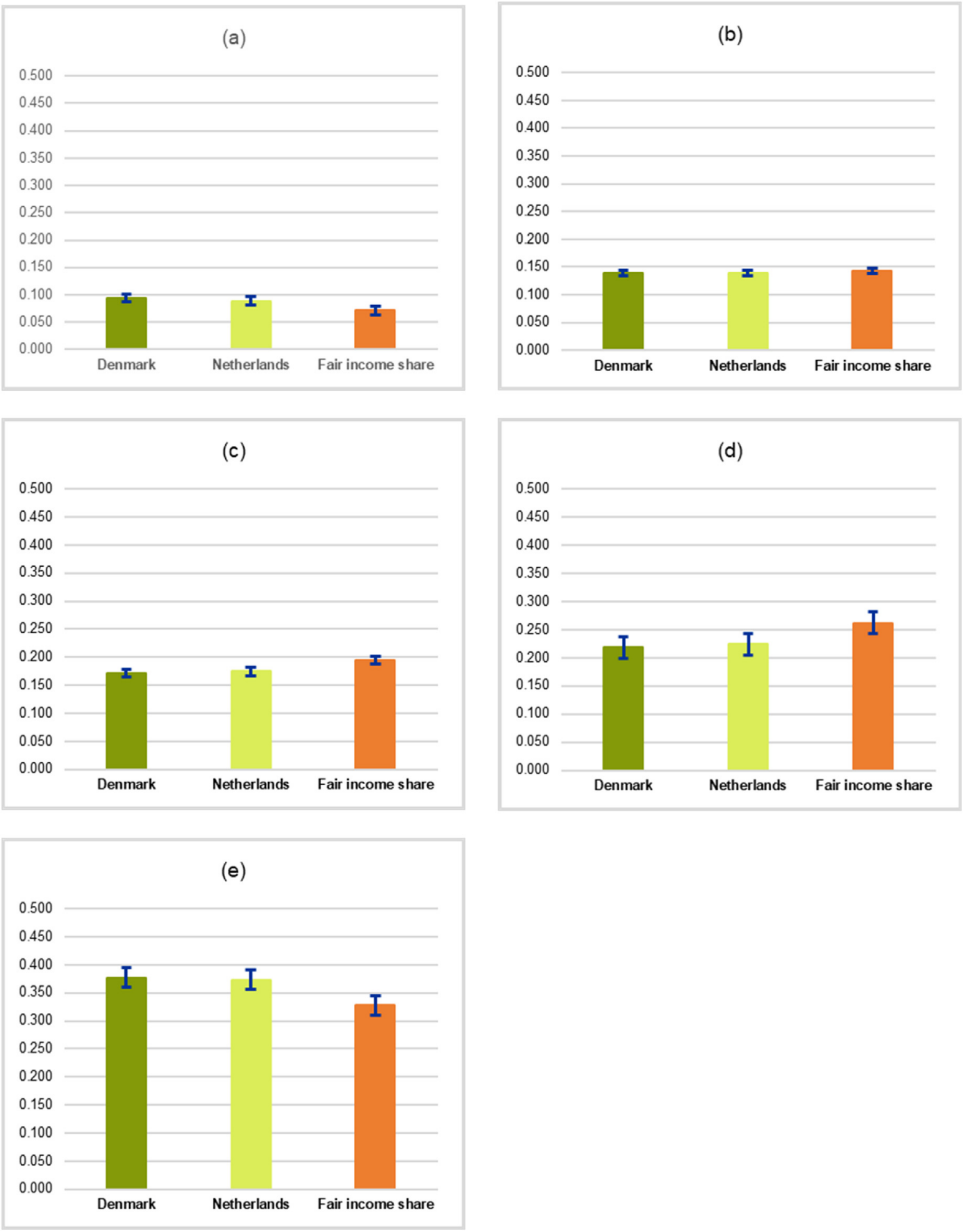
Actual income shares by quintile of Denmark and the Netherlands vs. fair income shares by quintile based on the professional athlete’s salary benchmark. (a) The lowest 20%. (b) The second 20%. (c) The third 20%. (d) The fourth (20%). (e) The top 20%. Note that the Gini index of Denmark and the Netherlands is equal to 0.282. The standard errors are indicated by the bars.

For the analysis of fair income distribution by quintile among different groups within a country and across countries, we choose Denmark and the Netherlands, on the criteria that both countries have the same Gini index (0.282) but differ in the income shares by quintile, in order to demonstrate that our method not only would be able to identify which quintile the income share of a country is fair or not fair, but also suggest the level of fair income shares by quintile for that country. In addition, the income distribution by quintile between these two countries could be compared since they are gauged using the same professional athlete’s salary benchmark. Our results are shown in Figure 7.

We can see from Figure 7 that both Denmark and the Netherlands share a common pattern of income shares by quintile (in decimals), only slightly differ in the degree of fairness. Given the existing value of the Gini index which is equal to 0.282 in both countries, our professional athlete’s salary benchmark indicates that the fair income share for the lowest 20% should be equal to 0.072 (Figure 7a). However, the actual income shares in the 1^st^ quintile of Denmark and the Netherlands are equal to 0.094 and 0.089, respectively. The fair income share for the second 20% should be 0.143 according to our professional athlete’ salary benchmark, but both Denmark and the Netherlands have slightly lower income shares in this quintile at 0.139 (Figure 7b). For the third 20%, the actual income share in Denmark equals 0.172 and that in the Netherlands equals 0.175 which are lower than the fair income share based on our professional athlete’s salary benchmark which suggests that it should equal 0.195 (Figure 7c). While our professional athlete’s salary benchmark indicates that the fair income share for the fourth 40% should equal 0.262, both Denmark and the Netherlands have lower actual income shares at 0.218 and 0.224, respectively (Figure 7d). For the top 20%, our professional athlete’s salary benchmark suggests that the fair income share should be 0.328 (Figure 7e). However, both Denmark and the Netherlands have higher levels of actual income shares at 0.377 and 0.373, respectively. Overall, given that both countries have the same Gini index, the Netherlands has a fairer income distribution than Denmark since the sum of the percentage deviation (in absolute value) of the actual quintile income shares from the corresponding fair quintile income shares consistent with the professional athlete’s salary benchmark of the Netherlands (65.08%) is less than that of Denmark (77.08%).

## 4 Discussion

Given that rising income inequality is a persistent cause of concern, the greater focus is needed to lower income inequality (the United Nations, 2019). While the SDG 10 (Target 10.1) calls for actions to reduce income inequality within and among countries by progressively achieving and sustaining income growth of the bottom 40% of the population at a rate higher than the national average by 2030, it should be aware that a low level of income inequality could potentially have adverse effects on economic development (Grigoli and Robles, 2017). Imagine a society where everyone has more or less similar income. In such a society, there should be no incentive for anyone to be creative or try to do things differently because no matter how hard they try or what they do and/or invent, there would be no extra benefits. This could put that society into an incentive trap which has negative impacts on productivity and economic growth (Sitthiyot and Holasut, 2016). In addition, contrary to the view that high income inequality is an ethical and moral concern across cultures around the world (the United Nations, 2020b), a wealth of empirical research in behavioral economics and psychology as discussed in this study has shown that people are not troubled by income inequality per se. Indeed, they prefer fair income distributions, not equal ones, both in laboratory conditions and in the real world (Starmans et al., 2017). People even support substantial income inequalities if they perceive as fair (Trump, 2020). Thus, from both theoretical and practical points of view, it is very important to know what level of inequality in income distribution is considered as fair.

In this study, we devise an alternative benchmark for measuring whether the existing level of income distribution of a country is fair or not, and if not, what fair income distribution in that country should be. Our benchmark is constructed based on the concepts of procedural justice, distributive justice, and power of authority in the realm of 11 well-known professional sports where the allocations of athlete’s salary are the outcomes of fair rules, individual and/or team performance, and luck. While the political economy of what levels of income differences countries would accept as fair remains an unexplored territory (Rogoff, 2012), with the exception of a few studies by Venkatasubramanian (2009, 2010, 2019) and Venkatasubramanian et al. (2015) who propose the use of entropy in statistical mechanics and information theory as a quantitative measure of fairness in income distribution and a study by Park and Kim (2021) who introduce the concept of a feasible income equality that maximizes the total social welfare and show that an optimal income distribution representing the feasible equality could be modeled by using the sigmoid welfare function and the Boltzmann income distribution, to our knowledge, no study has attempted to quantitatively measure fair income distribution across countries using the benchmark that is derived from the statistical relationship between the salary shares of the professional athlete in each quintile and the Gini index for the professional athlete’s salary in this way before. Our benchmark also satisfies the no-envy principle of fair allocation introduced by Tinbergen (1930) and the general consensus or the international norm criterion of a meaningful benchmark according to the OHCHR (2012). At the very least, the benchmark and the findings presented in our study could be viewed as another proof of concept that fair income distribution could be quantified. If future research could find other types of data that are fairer based on the concepts of procedural justice, distributive justice, and power of authority, and more universally accepted than the data on the distributions of athlete’s salary in professional sports employed in this study, our method could still be used to develop the fairness benchmarks, provided such data can be numerically ranked. This is because we can employ an appropriate functional form for the Lorenz curve to fit those data, calculate their respective values of the Gini index and the quantile shares, and find the fairness benchmarks by estimating the statistical relationships between the values of the Gini index and the same quantile shares across those data.

To demonstrate how our benchmark works, we use the data on the Gini index and the income shares by quintile in 2015 from the World Bank (2020) comprising 75 countries. The overall results indicate that the income earners in the lowest 20%, the second 20% (35 countries), and the top 20% in countries in the sample (except those in the lowest 20% in Romania) receive income shares higher than the fair shares according to our professional athlete’s salary benchmark, with those in the lowest 20% receive the highest share as measured by the maximum, the mean, and the median values of country’s income share. For those in the second 20% (40 countries), the third 20% and the fourth 20%, our results suggest that, the income earners in countries in the sample receive income shares lower than the fair shares based on the professional athlete’s salary benchmark, with those in the fourth 20% receive the lowest share as measured by the minimum, the mean, and the median values of country’s income share. By using Denmark and the Netherlands as examples, we demonstrate that our method could be used to compare fair income distribution among different groups within a country and across countries, provided that they share the same Gini index.

Furthermore, our professional athlete’s salary benchmark could be employed as a guideline for setting targets for fair income distribution as measured by the Gini index and the income shares by quintile. For example, supposing that an acceptable value of the Gini index is 0.226 and the minimum level of income share for the bottom 40% must be at least 25% of the total income share, our benchmark suggests that the fair income shares by quintile should be such that the top 20% and the fourth 20% should receive income shares around 29.4% and 25.3%, respectively. The third 20% would receive income share about 20.3% whereas the second 20% and the lowest 20% should receive income shares roughly 16.1% and 8.9%, respectively. Based on this example, the bottom 40% would receive income share around 25% and the income gap between the top 20% and the bottom 20% would be approximately 3.29 times. In the real world, there are infinite combinations of income shares by quintile that could yield the same value of the Gini index but there is only one of them that is regarded as fair. Thus, by using our benchmark, different countries could choose different combinations of the Gini index and the fair income shares by quintile that are appropriate for their own context before formulating and conducting policies in order to achieve the SDG 10 and other related SDGs.

While one of the major concerns about income inequality has been on the income earners whose income shares are in the bottom 40% (Keeley, 2015; the United Nations, 2015; the World Bank, 2015; the Organization for Economic Co-operation and Development (OECD), 2019; the United Nations, 2020a; the United Nations, 2020b; the World Bank, 2020b), our empirical findings from 75 countries indicate that, *on the fairness ground*, the majority of the income earners in the bottom 40% is in a relatively better position, as measured by the income shares they receive, than those in the third 20% and the fourth 20% who receive income shares lower than the fair shares. These results are well supported by the stylized facts known as “the Elephant Chart” published in Alvaredo et al. (2017, p. 13) and “the Loch Ness Monster Chart” published in Kharas and Seidel (2018, p. 12), both of which represent the relationship between the income growth and the quantile global income distribution. Both “the Elephant Chart” and “the Loch Ness Monster Chart”, according to these 2 studies, clearly illustrate that the income growth of the income earners in the third 20% and the fourth 20% is lower than that of the income earners in the other 3 quintiles. Another study by the OECD (2019) also finds similar results in that the middle-income households in the OECD countries have experienced dismal income growth or even stagnation in some countries. According to the OECD (2019), this has fueled perceptions that the current socio-economic system is *unfair* [emphasis in original] and that the middle class has not benefited from economic growth in proportion to its contribution. These empirical findings indicate that, in addition to the existing tools and/or diagrams used to study issues in income distribution, fairness and income inequality could be analyzed simultaneously through the lens of procedural justice, distributive justice, and power of authority using the fairness benchmark that is derived from the relationship between the salary shares of the professional athlete by quintile and the Gini index for the professional athlete’s salary.

Despite the empirical findings with regard to the level of fair income share of the bottom 40%, we would like to emphasize that this by no means implies that we should not continue to pursue policies in order to increase and sustain the income growth of the bottom 40% as stated in the Target 10.1 of the SDG 10. Reducing income inequality is found to be positively correlated with a number of socio-economic indicators that are fundamental for sustainable development (Wilkinson and Pickett, 2007). However, as noted by Frankfurt (2015) and Starmans et al. (2017), worries about income inequality are often conflated with worries about poverty and unfairness whereas these concerns should be thought of as distinct. According to Starmans et al. (2017), if income inequality in itself is not really what is bothering people as supported by laboratory and real-world studies, then we would be better off by clearly distinguishing these concerns, and shifting our attention to the problem that matters to us more which, in this case, is income inequity. After all, from the viewpoint of morality as argued by Frankfurt (2015, p. 7), it is not important that everyone should have *the same* [emphasis in original]. What is morally important is that everyone has *enough* [emphasis in original], provided that the income a person has results from fair processes and is proportional to his/her contributions. According to Frankfurt (2015, p. 7), if everyone has enough income, it should be of no special or deliberate concern whether some people have more income than others.

From our viewpoint, the grand challenge for everyone involved in formulating and implementing policies in order to achieve fair income distribution societies, the SGD 10, and other SDGs that are directly or indirectly related to the SDG 10 is not only to make processes with regard to the income allocations and the distributions of income fair but also to make the authorities being transparent and trustworthy when it comes to the issues of enacting rules, resolving disputes, and allocating resources. Moreover, people have to be educated and nudged so that they understand what make such processes and allocations fair. According to Dunham et al. (2018), this requires an understanding that a process can be fair despite producing an unequal outcome. It also requires understanding the concept of randomness since some processes can be fair by the virtue of being unpredictable and thereby not exploitable by involved parties. Furthermore, people themselves must be able to override their own selfishness and come to prefer processes that are procedurally fair and allocations that are distributively fair, even when these processes and allocations might materially put them in disadvantage positions. This is a tall order task. What forms of governance and institution that are capable of pulling this off and how long it would take remain unclear. In the meantime, we hope that other scientific disciplines could apply our method to analyze fair size distributions of resources and other non-negative quantities.

## Declarations

### Author contribution statement

Thitithep Sitthiyot: Conceived and designed the experiments; Performed the experiments; Analyzed and interpreted the data; Contributed reagents, materials, analysis tools or data; Wrote the paper.

Kanyarat Holasut: Analyzed and interpreted the data; Contributed reagents, materials, analysis tools or data; Wrote the paper.

### Funding statement

This research did not receive any specific grant from funding agencies in the public, commercial, or not-for-profit sectors.

### Data availability statement

All data generated and/or analyzed during this study are included in this article and can be accessed from the data repository and the World Bank as listed below.

1. Sitthiyot, T., 2021. Annual salaries of the athletes from 11 professional sports (V1). Mendeley Data. https://doi.org/10.17632/6pf936739y.1.

2. The World Bank, 2020a. World Bank Open Data. https://data.worldbank.org/.

### Declaration of interest’s statement

The authors declare no conflict of interest.

### Additional information

No additional information is available for this paper.

## Figures and Tables

**Table A1:** Actual income shares by quintile vs. fair income shares by quintile and the Gini index of 75 countries.

Country	Gini index	Income share	Top 20%	%Δ	Fourth 20%	%Δ	Third 20%	%Δ	Second 20%	%Δ	Lowest 20%	%Δ
1. Slovenia	0.254	Actual	0.351	13.18	0.226	-12.39	0.182	-8.80	0.145	-4.72	0.096	19.77
		Fair	0.310		0.258		0.200		0.152		0.080	
2. Ukraine	0.255	Actual	0.356	14.57	0.224	-13.22	0.178	-10.74	0.143	-5.84	0.100	25.24
		Fair	0.311		0.258		0.199		0.152		0.080	
3. Belarus	0.256	Actual	0.355	14.02	0.226	-12.50	0.180	-9.66	0.143	-5.65	0.097	21.95
		Fair	0.311		0.258		0.199		0.152		0.080	
4. Czech Republic	0.259	Actual	0.359	14.61	0.219	-15.37	0.178	-10.46	0.147	-2.39	0.097	23.38
		Fair	0.313		0.259		0.199		0.151		0.079	
5. Slovak Republic	0.265	Actual	0.350	10.40	0.232	-10.65	0.187	-5.47	0.146	-1.80	0.085	10.67
		Fair	0.317		0.260		0.198		0.149		0.077	
6. Kosovo	0.265	Actual	0.361	13.87	0.227	-12.58	0.177	-10.53	0.140	-5.84	0.095	23.69
		Fair	0.317		0.260		0.198		0.149		0.077	
7. Kazakhstan	0.268	Actual	0.369	15.69	0.222	-14.64	0.172	-12.84	0.137	-7.25	0.100	31.73
		Fair	0.319		0.260		0.197		0.148		0.076	
8. Moldova	0.270	Actual	0.369	15.22	0.223	-14.35	0.175	-11.17	0.137	-6.84	0.097	28.77
		Fair	0.320		0.260		0.197		0.147		0.075	
9. Finland	0.271	Actual	0.367	14.37	0.224	-14.01	0.175	-11.09	0.140	-4.59	0.094	25.28
		Fair	0.321		0.260		0.197		0.147		0.075	
10. Norway	0.275	Actual	0.365	12.82	0.227	-13.03	0.177	-9.76	0.141	-3.06	0.090	21.84
		Fair	0.324		0.261		0.196		0.145		0.074	
11. Belgium	0.277	Actual	0.365	12.36	0.227	-13.12	0.181	-7.56	0.140	-3.31	0.086	17.34
		Fair	0.325		0.261		0.196		0.145		0.073	
12. Denmark	0.282	Actual	0.377	14.87	0.218	-16.76	0.172	-11.75	0.139	-2.90	0.094	30.80
		Fair	0.328		0.262		0.195		0.143		0.072	
13. Netherlands	0.282	Actual	0.373	13.65	0.224	-14.46	0.175	-10.21	0.139	-2.90	0.089	23.84
		Fair	0.328		0.262		0.195		0.143		0.072	
14. Kyrgyz Republic	0.290	Actual	0.388	16.29	0.215	-18.18	0.168	-13.15	0.134	-4.64	0.095	36.42
		Fair	0.334		0.263		0.193		0.141		0.070	
15. Sweden	0.292	Actual	0.376	12.23	0.228	-13.30	0.176	-8.83	0.139	-0.61	0.082	18.69
		Fair	0.335		0.263		0.193		0.140		0.069	
16. Malta	0.294	Actual	0.381	13.25	0.225	-14.50	0.175	-9.17	0.134	-3.73	0.085	24.01
		Fair	0.336		0.263		0.193		0.139		0.069	
17. Hungary	0.304	Actual	0.384	11.78	0.229	-13.27	0.176	-7.71	0.133	-2.11	0.078	18.41
		Fair	0.344		0.264		0.191		0.136		0.066	
18. Austria	0.305	Actual	0.384	11.54	0.229	-13.29	0.176	-7.61	0.132	-2.60	0.079	20.41
		Fair	0.344		0.264		0.191		0.136		0.066	
19 Croatia	0.311	Actual	0.384	10.14	0.235	-11.16	0.178	-5.95	0.131	-1.88	0.073	13.96
		Fair	0.349		0.265		0.189		0.134		0.064	
20. Germany	0.317	Actual	0.397	12.44	0.226	-14.68	0.170	-9.57	0.129	-1.90	0.078	24.73
		Fair	0.353		0.265		0.188		0.131		0.063	
21. Egypt, Arab Rep.	0.318	Actual	0.415	17.29	0.206	-22.25	0.160	-14.80	0.128	-2.41	0.091	46.10
		Fair	0.354		0.265		0.188		0.131		0.062	
22. Ireland	0.318	Actual	0.402	13.61	0.221	-16.58	0.169	-10.00	0.129	-1.64	0.080	28.44
		Fair	0.354		0.265		0.188		0.131		0.062	
23. Poland	0.318	Actual	0.392	10.78	0.231	-12.81	0.175	-6.81	0.128	-2.41	0.074	18.81
		Fair	0.354		0.265		0.188		0.131		0.062	
24. Switzerland	0.323	Actual	0.402	12.41	0.226	-14.77	0.168	-10.01	0.125	-3.45	0.078	27.78
		Fair	0.358		0.265		0.187		0.129		0.061	
25. Armenia	0.324	Actual	0.407	13.57	0.218	-17.80	0.167	-10.44	0.126	-2.42	0.082	34.88
		Fair	0.358		0.265		0.186		0.129		0.061	
26. Estonia	0.327	Actual	0.404	12.01	0.232	-12.56	0.164	-11.74	0.125	-2.43	0.075	24.87
		Fair	0.361		0.265		0.186		0.128		0.060	
27. France	0.327	Actual	0.409	13.40	0.217	-18.21	0.167	-10.12	0.128	-0.09	0.079	31.53
		Fair	0.361		0.265		0.186		0.128		0.060	
28. Tunisia	0.328	Actual	0.409	13.15	0.225	-15.21	0.165	-11.09	0.123	-3.74	0.078	30.39
		Fair	0.361		0.265		0.186		0.128		0.060	
29. United Kingdom	0.332	Actual	0.406	11.37	0.230	-13.36	0.168	-9.03	0.122	-3.49	0.075	27.42
		Fair	0.365		0.265		0.185		0.126		0.059	
30. Pakistan	0.335	Actual	0.428	16.65	0.205	-22.80	0.156	-15.22	0.122	-2.71	0.089	53.06
		Fair	0.367		0.266		0.184		0.125		0.058	
31. Luxembourg	0.338	Actual	0.410	11.02	0.231	-13.02	0.167	-8.89	0.121	-2.72	0.072	25.34
		Fair	0.369		0.266		0.183		0.124		0.057	
32. Cyprus	0.340	Actual	0.421	13.51	0.217	-18.30	0.162	-11.39	0.121	-2.18	0.079	38.65
		Fair	0.371		0.266		0.183		0.124		0.057	
33. Tajikistan	0.340	Actual	0.417	12.43	0.224	-15.66	0.164	-10.30	0.120	-2.99	0.074	29.88
		Fair	0.371		0.266		0.183		0.124		0.057	
34. Latvia	0.342	Actual	0.415	11.41	0.228	-14.16	0.164	-10.07	0.123	-0.02	0.071	25.64
		Fair	0.372		0.266		0.182		0.123		0.057	
35. Ethiopia	0.350	Actual	0.430	13.46	0.212	-20.18	0.163	-9.67	0.121	0.58	0.073	33.48
		Fair	0.379		0.266		0.180		0.120		0.055	
36. Italy	0.354	Actual	0.413	8.04	0.235	-11.50	0.172	-4.16	0.121	1.74	0.059	9.67
		Fair	0.382		0.266		0.179		0.119		0.054	
37. Portugal	0.355	Actual	0.427	11.46	0.223	-16.01	0.163	-9.05	0.120	1.19	0.067	25.05
		Fair	0.383		0.266		0.179		0.119		0.054	
38. North Macedonia	0.356	Actual	0.411	7.05	0.242	-8.85	0.174	-2.77	0.117	-1.06	0.056	4.95
		Fair	0.384		0.265		0.179		0.118		0.053	
39. Gambia, The	0.359	Actual	0.436	12.82	0.218	-17.86	0.157	-11.90	0.116	-1.05	0.074	40.41
		Fair	0.386		0.265		0.178		0.117		0.053	
40. Romania	0.359	Actual	0.407	5.32	0.247	-6.93	0.177	-0.68	0.118	0.66	0.051	-3.23
		Fair	0.386		0.265		0.178		0.117		0.053	
41. Greece	0.360	Actual	0.418	7.93	0.235	-11.44	0.170	-4.48	0.118	0.95	0.059	12.41
		Fair	0.387		0.265		0.178		0.117		0.052	
42. Thailand	0.360	Actual	0.438	13.09	0.219	-17.47	0.155	-12.90	0.113	-3.33	0.075	42.90
		Fair	0.387		0.265		0.178		0.117		0.052	
43. Spain	0.362	Actual	0.421	8.23	0.235	-11.42	0.170	-4.20	0.117	0.68	0.058	11.43
		Fair	0.389		0.265		0.177		0.116		0.052	
44. Georgia	0.365	Actual	0.434	10.85	0.226	-14.78	0.159	-10.01	0.113	-1.90	0.067	30.33
		Fair	0.392		0.265		0.177		0.115		0.051	
45. Lithuania	0.374	Actual	0.441	10.45	0.221	-16.51	0.159	-8.81	0.116	3.45	0.061	23.18
		Fair	0.399		0.265		0.174		0.112		0.050	
46. Tonga	0.376	Actual	0.454	13.21	0.212	-19.87	0.152	-12.56	0.114	2.29	0.068	38.47
		Fair	0.401		0.265		0.174		0.111		0.049	
47. Russian Federation	0.377	Actual	0.453	12.71	0.215	-18.72	0.152	-12.43	0.111	-0.10	0.069	41.10
		Fair	0.402		0.265		0.174		0.111		0.049	
48. Myanmar	0.381	Actual	0.457	12.72	0.206	-22.04	0.150	-13.04	0.113	2.96	0.073	51.80
		Fair	0.405		0.264		0.172		0.110		0.048	
49. China	0.386	Actual	0.454	10.76	0.223	-15.47	0.153	-10.60	0.106	-1.90	0.064	35.91
		Fair	0.410		0.264		0.171		0.108		0.047	
50. Iran, Islamic Rep.	0.395	Actual	0.464	10.99	0.216	-17.85	0.150	-11.07	0.107	1.90	0.063	38.97
		Fair	0.418		0.263		0.169		0.105		0.045	
51. Serbia	0.396	Actual	0.444	5.97	0.233	-11.35	0.165	-2.01	0.110	5.09	0.048	6.33
		Fair	0.419		0.263		0.168		0.105		0.045	
52. Indonesia	0.397	Actual	0.473	12.65	0.206	-21.59	0.146	-13.15	0.106	1.60	0.070	55.73
		Fair	0.420		0.263		0.168		0.104		0.045	
53. Uruguay	0.402	Actual	0.461	8.59	0.225	-14.17	0.154	-7.61	0.104	1.32	0.056	27.26
		Fair	0.425		0.262		0.167		0.103		0.044	
54. El Salvador	0.406	Actual	0.472	10.21	0.214	-18.20	0.148	-10.60	0.105	3.65	0.061	41.01
		Fair	0.428		0.262		0.166		0.101		0.043	
55. Kenya	0.408	Actual	0.475	10.43	0.215	-17.74	0.146	-11.50	0.103	2.35	0.062	44.55
		Fair	0.430		0.261		0.165		0.101		0.043	
56. Malaysia	0.410	Actual	0.473	9.48	0.220	-15.74	0.148	-9.97	0.101	1.04	0.058	36.39
		Fair	0.432		0.261		0.164		0.100		0.043	
57. Cote d'Ivoire	0.415	Actual	0.478	9.43	0.216	-17.04	0.146	-10.40	0.102	3.77	0.057	36.95
		Fair	0.437		0.260		0.163		0.098		0.042	
58. Turkey	0.429	Actual	0.492	9.24	0.211	-18.22	0.142	-10.56	0.099	5.73	0.056	42.95
		Fair	0.450		0.258		0.159		0.094		0.039	
59. Togo	0.431	Actual	0.486	7.43	0.224	-13.06	0.144	-8.95	0.095	2.17	0.050	28.75
		Fair	0.452		0.258		0.158		0.093		0.039	
60. Peru	0.434	Actual	0.487	6.95	0.218	-15.20	0.148	-5.88	0.099	7.62	0.047	22.62
		Fair	0.455		0.257		0.157		0.092		0.038	
61. Philippines	0.444	Actual	0.509	9.37	0.207	-18.84	0.135	-12.43	0.093	4.83	0.057	55.39
		Fair	0.465		0.255		0.154		0.089		0.037	
62. Dominican Republic	0.452	Actual	0.511	7.90	0.209	-17.48	0.138	-9.00	0.093	7.99	0.050	41.22
		Fair	0.474		0.253		0.152		0.086		0.035	
63. Ecuador	0.460	Actual	0.513	6.46	0.213	-15.25	0.138	-7.45	0.091	8.93	0.046	34.63
		Fair	0.482		0.251		0.149		0.084		0.034	
64. Bolivia	0.467	Actual	0.511	4.45	0.218	-12.63	0.142	-3.30	0.090	10.69	0.039	17.79
		Fair	0.489		0.250		0.147		0.081		0.033	
65. Paraguay	0.476	Actual	0.527	5.65	0.209	-15.40	0.134	-6.88	0.087	10.88	0.043	35.26
		Fair	0.499		0.247		0.144		0.078		0.032	
66. Chile	0.477	Actual	0.536	7.23	0.197	-20.17	0.130	-9.45	0.089	13.89	0.048	51.68
		Fair	0.500		0.247		0.144		0.078		0.032	
67. Benin	0.478	Actual	0.521	4.00	0.208	-15.61	0.142	-0.87	0.096	23.34	0.032	1.58
		Fair	0.501		0.246		0.143		0.078		0.032	
68. Costa Rica	0.484	Actual	0.539	6.22	0.206	-15.82	0.128	-9.38	0.084	10.58	0.043	40.29
		Fair	0.507		0.245		0.141		0.076		0.031	
69. Honduras	0.496	Actual	0.540	3.73	0.214	-11.18	0.132	-3.81	0.078	7.94	0.036	24.12
		Fair	0.521		0.241		0.137		0.072		0.029	
70. Panama	0.508	Actual	0.553	3.57	0.205	-13.45	0.128	-3.86	0.080	16.56	0.034	23.97
		Fair	0.534		0.237		0.133		0.069		0.027	
71. Colombia	0.511	Actual	0.559	4.03	0.200	-15.18	0.125	-5.38	0.079	16.63	0.038	40.53
		Fair	0.537		0.236		0.132		0.068		0.027	
72. Brazil	0.513	Actual	0.561	3.97	0.197	-16.19	0.127	-3.36	0.079	17.66	0.036	34.39
		Fair	0.540		0.235		0.131		0.067		0.027	
73. Botswana	0.533	Actual	0.585	4.00	0.195	-14.25	0.111	-10.81	0.070	14.18	0.039	60.19
		Fair	0.562		0.227		0.124		0.061		0.024	
74. Zambia	0.571	Actual	0.613	0.93	0.193	-8.42	0.106	-4.42	0.060	18.03	0.029	43.80
		Fair	0.607		0.211		0.111		0.051		0.020	
75. Namibia	0.591	Actual	0.637	0.87	0.179	-10.92	0.098	-5.46	0.058	26.97	0.028	53.95
		Fair	0.632		0.201		0.104		0.046		0.018	

Note: The Gini index and income shares are shown in decimals.
